# Volumetric analysis of hippocampal subregions and subfields in left and right semantic dementia

**DOI:** 10.1093/braincomms/fcae097

**Published:** 2024-03-25

**Authors:** Arenn F Carlos, Stephen D Weigand, Joseph R Duffy, Heather M Clark, Rene L Utianski, Mary M Machulda, Hugo Botha, Nha Trang Thu Pham, Val J Lowe, Christopher G Schwarz, Jennifer L Whitwell, Keith A Josephs

**Affiliations:** Department of Neurology, Mayo Clinic, Rochester, MN 55905 USA; Department of Quantitative Health Sciences, Mayo Clinic, Rochester, MN 55905 USA; Department of Neurology, Mayo Clinic, Rochester, MN 55905 USA; Department of Neurology, Mayo Clinic, Rochester, MN 55905 USA; Department of Neurology, Mayo Clinic, Rochester, MN 55905 USA; Department of Psychiatry and Psychology, Mayo Clinic, Rochester, MN 55905 USA; Department of Neurology, Mayo Clinic, Rochester, MN 55905 USA; Department of Radiology, Mayo Clinic, Rochester, MN 55905 USA; Department of Radiology, Mayo Clinic, Rochester, MN 55905 USA; Department of Radiology, Mayo Clinic, Rochester, MN 55905 USA; Department of Radiology, Mayo Clinic, Rochester, MN 55905 USA; Department of Neurology, Mayo Clinic, Rochester, MN 55905 USA

**Keywords:** anterior temporal pole, hippocampal segmentation, right temporal semantic dementia, semantic variant PPA, temporopolar syndromes

## Abstract

Two variants of semantic dementia are recognized based on the laterality of temporal lobe involvement: a left-predominant variant associated with verbal knowledge impairment and a right-predominant variant associated with behavioural changes and non-verbal knowledge loss. This cross-sectional clinicoradiologic study aimed to assess whole hippocampal, subregion, and/or subfield volume loss in semantic dementia versus controls and across its variants. Thirty-five semantic dementia participants and 15 controls from the Neurodegenerative Research Group at Mayo Clinic who had completed 3.0-T volumetric magnetic resonance imaging and ^18^F-fluorodeoxyglucose-positron emission tomography were included. Classification as left-predominant (*n* = 25) or right-predominant (*n* = 10) variant was based on temporal lobe hypometabolism. Volumes of hippocampal subregions (head, body, and tail) and subfields (parasubiculum, presubiculum, subiculum, *cornu ammonis* 1, *cornu ammonis* 3, *cornu ammonis* 4, dentate gyrus, molecular layer, hippocampal–amygdaloid transition area, and fimbria) were obtained using FreeSurfer 7. Subfield volumes were measured separately from head and body subregions. We fit linear mixed-effects models using log-transformed whole hippocampal/subregion/subfield volumes as dependent variables; age, sex, total intracranial volume, hemisphere and a group-by-hemisphere interaction as fixed effects; and subregion/subfield nested within hemisphere as a random effect. Significant results (*P* < 0.05) are hereby reported. At the whole hippocampal level, the dominant (predominantly involved) hemisphere of both variants showed 23–27% smaller volumes than controls. The non-dominant (less involved) hemisphere of the right-predominant variant also showed volume loss versus controls and the left-predominant variant. At the subregional level, both variants showed 17–28% smaller dominant hemisphere head, body, and tail than controls, with the right-predominant variant also showing 8–12% smaller non-dominant hemisphere head than controls and left-predominant variant. At the subfield level, the left-predominant variant showed 12–36% smaller volumes across all dominant hemisphere subfields and 14–15% smaller non-dominant hemisphere parasubiculum, presubiculum (head and body), subiculum (head) and hippocampal–amygdaloid transition area than controls. The right-predominant variant showed 16–49% smaller volumes across all dominant hemisphere subfields and 14–22% smaller parasubiculum, presubiculum, subiculum, *cornu ammonis* 3, hippocampal–amygdaloid transition area (all from the head) and fimbria of non-dominant hemisphere versus controls. Comparison of dominant hemispheres showed 16–29% smaller volumes of the parasubiculum, presubiculum (head) and fimbria in the right-predominant than left-predominant variant; comparison of non-dominant hemispheres showed 12–15% smaller *cornu ammonis* 3, *cornu ammonis* 4, dentate gyrus, hippocampal–amygdaloid transition area (all from the head) and *cornu ammonis* 1, *cornu ammonis 3* and *cornu ammonis* 4 (all from the body) in the right-predominant variant. All hippocampal subregion/subfield volumes are affected in semantic dementia, although some are more affected in both dominant and non-dominant hemispheres of the right-predominant than the left-predominant variant by the time of presentation. Involvement of hippocampal structures is apparently more subregion dependent than subfield dependent, indicating possible superiority of subregion volumes as disease biomarkers.

## Teaser

Carlos *et al*. studied the changes in hippocampal subregion and subfield volumes in semantic dementia and its left and right variants. All hippocampal subregions/subfields showed volume loss in semantic dementia, with the right-predominant variant showing more bilateral involvement at presentation. Volume loss appeared to be more subregion dependent than subfield dependent.

## Graphical Abstract

**Graphical Abstract:**
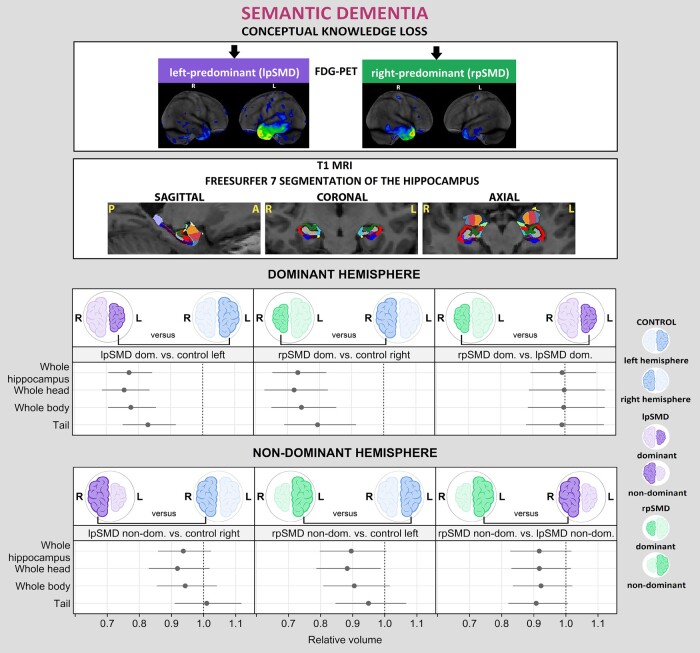

## Introduction

According to Tulving,^1,2^ semantic memory is a system for receiving, retaining and transmitting information about the knowledge and classification of concepts, facts, objects and their relations, as well as that of words and their meaning, that are unconnected to specific temporal and/or spatial experiences (versus episodic memory). Cases with symptoms suggestive of impaired semantic memory can be traced back to more than a century ago with Arnold Pick’s original cases of focal dementias^3^ or the *Gogi* aphasia recognized in Japan beginning early 1900s.^4^ In the English literature, Warrington^5^ first reported in 1975 the selective impairment of semantic memory in patients with object and word agnosia. The term semantic dementia (SMD) was later coined by Snowden *et al.*^6^ and endorsed by Hodges *et al.*^7^ to describe a degenerative disorder of the anterior temporal lobes, manifesting as profound impairment in naming and comprehension due to loss of conceptual knowledge involving both verbal and non-verbal domains. Since then, SMD has been expanded to include conceptual knowledge loss involving numerous types of sensory domains, with failure to recognize visual stimuli, including faces (prosopagnosia)^8-12^ and environmental landmarks (topographagnosia);^9^ auditory stimuli, including voices,^12,13^ environmental sounds,^14-16^ and melodies;^15^ olfactory stimuli such as odors;^17^ gustatory stimuli such as flavours or tastes;^18,19^ and tactile stimuli, including pain and temperature sensations.^20,21^

Neuroimaging studies in patients with SMD have consistently found focal involvement of the temporal lobes with different types of structural and functional neuroimaging. More specifically, the temporal pole and the inferior and middle temporal gyri,^22-25^ as well as the medial temporal lobe structures and the fusiform gyrus are typically involved.^9,23,26^ Most neuroimaging studies have shown bilateral, often strikingly asymmetric, degeneration of the anteromedial temporal lobes in SMD.^7,23,27^ Some studies have also found unilateral involvement, particularly in the early stages of the disease, which over time progressed to also affect the contralateral hemisphere.^26,28^ Depending on the laterality of the hemisphere/temporal lobe predominantly affected, two variants of SMD have been recognized: a left-predominant SMD (lpSMD) variant (also referred to as the semantic variant of primary progressive aphasia or svPPA) associated with impairment of language functions, including naming and impaired single word comprehension, as well as loss of word and object knowledge; and a right-predominant SMD (rpSMD) variant associated with behavioural changes, loss of insight and empathy, and impairment of non-verbal domains with frequent prosopagnosia and topographagnosia.^8,9,22,26,27,29-33^

One of the cardinal features of SMD is the relative sparing of episodic memory (particularly at early stages),^7,10^ which originally helped distinguish SMD from the typical Alzheimer’s type dementia at a time when Alzheimer’s dementia was often confused with frontotemporal dementias. Nonetheless, clinicoradiologic studies have consistently found the involvement of the hippocampus in SMD.^23,26,34-36^ Whilst the relationship between hippocampus and episodic memory is widely acknowledged, its association with semantic memory is less established. Studies have shown that the hippocampus also participates in semantic memory acquisition, maintenance, and real-time processing.^37,38^

To understand the functional roles of the hippocampus, knowledge of its anatomy is crucial. Along its longitudinal axis, the hippocampus can be divided into subregions, consisting of the anterior (or head) and posterior hippocampus, with the posterior hippocampus often further split into body and tail subregions.^39-41^ Along its transverse axis, the hippocampus is divided into anatomically and functionally distinct subfields, which grossly include the *cornu ammonis* (CA) regions, dentate gyrus, and subiculum.^40,42^ It is believed that differential functional specialization of the hippocampus and its substructures exists according to laterality (left or right), longitudinal axis (subregions) and transverse axis (subfields) orientation. The left hippocampus has been linked to verbal memory and is thus easily activated by words, whilst the right hippocampus is related to spatial and navigational (topographical) memory and is more activated by objects, pictures or scenes, and faces.^43-48^ Moreover, varying relationships of head and body subregions to other brain structures affect their roles in memory processing, such that the head subregion (which is connected to frontal and anterior/middle temporal regions) is involved in verbal, emotional, course gist-like and semantic memory, whereas the body and tail (which are connected to the parietooccipital and basal ganglia regions) are associated with spatial, navigational, and detailed episodic memory.^34,39,40,45,48-53^ Furthermore, the head subregion is more active during memory encoding, whilst the body and tail are more operational during retrieval.^39,40,48^ Across the subfields, CA1 and subiculum are found to associate with verbal and visual memory and appear to be more active during retrieval,^54^ whilst CA3 and the dentate gyrus are linked not only to verbal and visual memory but also to object recognition and pattern separation and are active during both encoding and retrieval.^40,54-56^ Only a few studies with relatively small numbers of svPPA or overall SMD patients (without variant distinction) have investigated the changes in the main hippocampal subregions or subfields in SMD patients relative to controls,^57-59^ and only one has separated SMD into left and right variants.^60^ Little remains known about the pattern of volume changes in both subregions and subfields in SMD, as well as between the two SMD variants and controls. Furthermore, no study has directly compared both the predominantly affected and the relatively spared hemispheres between the two SMD variants and none have investigated the subfields split by subregion localization.

In the present study, our primary aim was to determine whether whole hippocampal, subregion, and/or subfield volumes differed between SMD variants (lpSMD and rpSMD) and healthy controls, and whether any such differences would be restricted to the dominant (or predominantly affected) hemisphere or extend to involve the non-dominant (or relatively spared/less affected) hemisphere. As a secondary aim, we sought to directly compare subregion and subfield volumes between the two SMD variants and determine again whether differences would be more prominent in the dominant hemisphere compared with the non-dominant hemisphere. Given the above-mentioned premises, we hypothesized that the left hippocampus would show more volume loss in lpSMD, whilst the right hippocampus would be more affected in rpSMD compared with controls; that the anterior and, to a lesser extent, the posterior subregions of the hippocampus, along with their subfields, would have smaller volumes in the dominant hemispheres of both lpSMD and rpSMD relative to controls; and that the anterior subregion with its subfields would be more affected in the lpSMD group, whilst the posterior subregion and its subfields would be more affected in the rpSMD group.

## Materials and methods

### Design, setting and participants

This cross-sectional clinicoradiologic study was conducted at Mayo Clinic in Rochester, MN, USA. All study participants had been prospectively recruited into one of two National Institute of Health-funded grants (PI: K.A.J.) and followed by the Neurodegenerative Research Group between August 2011 and May 2023. All participants underwent standardized neurological, neuropsychological, speech-language, and neuroimaging evaluations. A total of 40 participants who had received a diagnosis of ‘SMD’ from a board-certified behavioural neurologist (K.A.J.) based on clinical presentation and neuroimaging features were identified. Only participants who underwent both a 3.0-T volumetric magnetic resonance imaging (MRI) and an ^18^F-fluorodeoxyglucose (FDG)-positron emission tomography (PET) were included in this study. All MRI and FDG-PET scans analysed for this study were from the first (baseline) visit. All scans were manually quality controlled by a neuroimaging analyst (N.T.T.P.) to access for segmentation errors. Of the 40 SMD participants, 37 passed quality control with 36 segmentations considered ‘excellent’ and one considered ‘good’. Three segmentations were considered ‘poor’. These participants had severely atrophic hippocampi and hence failed segmentation prior to analysis and were thus excluded. Additional two cases that passed quality control were also excluded due to hippocampal volume-temporal hypometabolism mismatch (i.e. showing more hypometabolism of the temporal lobe on one side yet appearing to have more volume loss on the other side). Therefore, a total of 35 SMD participants were included for this study. All 35 SMD participants were categorized into a lpSMD or rpSMD variant based on the laterality of the hemisphere with the greatest/most severe hypometabolism observed on FDG-PET (see ‘FDG-PET and SMD variants’ section below). In addition, 15 healthy controls closest in age to the SMD participants and who completed identical 3.0-T MRI brain scanning were included. The control group had a median Montreal Cognitive Assessment score of 26/30 and 67% were female.

This study was conducted in accordance with the Declaration of Helsinki and was approved by the Mayo Clinic Institutional Review Board. All participants or their proxies had signed written informed consent forms.

### Clinical diagnosis

Participants underwent standardized neurological, neuropsychological, and speech-language evaluations. The neurological examination consisted of an evaluation by a behavioural neurologist (K.A.J.) tailored to determine the presence of core and supportive clinical symptoms of SMD based on published criteria for SMD by Neary *et al*.^10^ and published criteria for the diagnosis of svPPA by Gorno-Tempini *et al.*^29^ The diagnosis, however, also warranted concomitant neuroimaging evidence of relatively focal anteromedial temporal lobe atrophy or hypometabolism in a ‘boxing glove’ pattern. Standardized tests performed during the neurological evaluation included the Frontal Assessment Battery (FAB)^61^ to assess frontal lobe functions; the Movement Disorder Society-sponsored revision of the Unified Parkinson’s Disease Rating scale (MDS-UPDRS)^62^ part III to evaluate parkinsonism and gait disturbances; the Western Aphasia Battery (WAB) apraxia subtest^63^ to test for ideomotor apraxia; and the Neuropsychiatric Inventory (NPI),^64^ the Cambridge Behavioural Inventory revised (CBI-R)^65^ and the 20-item Behavioural Assessment Scale (BAS)^66^ to appraise neurobehavioural and psychiatric symptoms. Moreover, medical records of participants were also reviewed for medications taken within a year prior to the research visit that could potentially affect (negatively or positively) cognitive performance or imaging findings. The neuropsychological battery included the Montreal Cognitive Assessment (MoCA)^67^ to screen for overall cognitive impairment; an in-house famous faces test to assess for prosopagnosia;^68^ an in-house auditory agnosia battery to test for recognition of non-verbal sounds and noises; and the word-picture version of the Pyramids and Palm Trees Test^69^ to evaluate for associative semantic knowledge. The speech-language evaluations included the administration of the Western Aphasia Battery spontaneous speech, auditory verbal comprehension, repetition, naming and word finding subtests to calculate an Aphasia Quotient (WAB-AQ),^63^ which measures language ability; the Sydney Language Battery (SYDBAT)^70^ naming and semantic association subtests; the F-A-S Letter Fluency test^71^ to test for phonemic word fluency; and the Boston Diagnostic Aphasia Examination (BDAE)^72^ repetition subtest. Finally, consensus meetings were held amongst neurologists, neuropsychologist, speech-language pathologists and neuroradiologists to obtain a final clinical diagnosis following consideration of all available clinical data.

### FDG-PET and SMD variants

The FDG-PET scans were acquired using a PET/CT scanner (GE Healthcare, Milwaukee, WI, USA) operating in 3D mode. Prior to injection, all participants were required to fast for a period of six hours. Participants were injected with ^18^F-FDG of ∼459 MBq (range 367–576 MBq). After injection, all participants were placed in a dimly lit room for 30 minutes, with minimal auditory stimulation. After the 30 minutes, an 8-minute scan was performed. FDG-PET consisted of four 2-minute dynamic frames acquired from 30 to 38 minutes after injection. PET sinograms were iteratively reconstructed into a 256-mm field of view (FOV). The pixel size was 1.0 mm and the slice thickness was 3.3 mm. Standard corrections were applied. Individual-level patterns of hypometabolism were assessed using a 3D stereotactic surface projections with CortexID Suite (GE Healthcare), whereby activity at each voxel was normalized to the pons and *Z*-scored to an age-segmented normative database. Average medial temporal and lateral temporal *Z*-scores (with negative scores representing hypometabolism) were calculated and compared between the left and right hemispheres. Participants were assigned to the lpSMD or rpSDM group based on the finding of at least 0.5 differences in medial and/or lateral temporal *Z*-scores between the left and right hemispheres.

### MRI acquisition

All participants underwent volumetric T_1_-weighted MRI on a 3.0-T GE scanner using a protocol that included a 3D magnetization-prepared rapid acquisition gradient echo (MPRAGE) performed using the following parameters: repetition time (TR)/echo time (TE)/inversion time (TI), 2300/3/900 ms; 8° flip angle; 1.2-mm slice thickness, 26-cm FOV; and 256 × 256 in-plane matrix (voxel size = 260/256 mm × 260/256 mm × 1.2 mm = 1.2 mm^3^).

### Hippocampal subregion and subfield volume analysis

The hippocampal subregions and subfields were segmented using FreeSurfer version 7.0 (Harvard Medical School, Boston, USA, https://surfer.nmr.mgh.harvard.edu/), which uses a probabilistic atlas built with ultrahigh-resolution *ex vivo* MRI data to produce an automated segmentation of the hippocampal substructures and amygdaloid nuclei.^73^ Structural T_1_-weighted MRI scans of participants were processed with the main FreeSurfer stream ‘recon-all’ pipeline. Volumes of the whole hippocampus, the subregions (head, body, and tail) and the subfields with head/body subdivision were obtained. The subfields of interests were parasubiculum, presubiculum, subiculum, CA1, CA3 (which also includes CA2), CA4, GC-ML-DG (dentate gyrus), molecular layer, hippocampal–amygdaloid transition area (HATA) and fimbria. Hippocampal substructure segmentations and the corresponding images were visually inspected using FreeSurfer’s Freeview tool to ensure appropriate segmentation. To allow correction for head size, total intracranial volumes were also calculated using the standard FreeSurfer processing pipeline.

### Statistical analyses

Participants were grouped into controls, lpSMDs, and rpSMDs. All statistical analyses were performed using R version 4.1.3. Significance level was set at *α* = 0.05. Categorical variables were described as counts and percentages, whilst continuous variables were described as medians and quartiles (Q1, Q3). Demographic, clinical, and neuroimaging data were compared amongst the three groups using Fisher’s exact test or Kruskal–Wallis test (or Wilcoxon rank-sum test, as appropriate).

Whole hippocampal volumes were defined as the sum of the head, body, and tail subregion volumes. Whole head and whole body subregion volumes were defined as the sum of all subfield volumes (presubiculum, subiculum, CA1, CA3, CA4, dentate gyrus and molecular layer) located in the head and body subregions, respectively. Following anatomical representations, the parasubiculum and the HATA were additionally included in the head subregion, whilst the fimbria was included in the body subregion. We referred to the predominantly affected hemisphere in SMD variants as the ‘dominant hemisphere’, whilst the spared hemisphere was designated ‘non-dominant hemisphere’. To compare SMD participants to controls, volumes from the dominant and the non-dominant hemispheres were compared with volumes from the corresponding left or right hemisphere in controls. Specifically, volumes from the lpSMD dominant (left) hemisphere were compared with volumes from control left hemisphere and volumes from the lpSMD non-dominant (right) hemisphere were compared with volumes from the control right hemisphere. A similar approach was used for comparison between rpSMD and controls. To assess differences between the SMD variants, hippocampal volumes from the dominant hemispheres and volumes from the non-dominant hemispheres of lpSMD and rpSMD were contrasted.

In total, we fit three linear mixed-effects models: the first model was a single-region analysis of the left and right whole hippocampal volumes; the second model was a three-region model at the subregion level, incorporating the whole hippocampal head, whole hippocampal body, and tail; and the third model was an 18-region model using the individual subfield volumes. For each model, log-transformed volume was the dependent variable and age, sex, total intracranial volumes, hemisphere and a group-by-hemisphere interaction were included as fixed effects. Random effects were used to obtain group-specific estimates for whole hippocampal, subregion and subfield volumes in each hemisphere. The models also included a random intercept for each hemisphere for each participant. Model estimation was via a Bayesian hierarchical approach using the R package *rstanarm* with default priors. Group-wise differences were exponentiated and the ratio of geometric means was interpreted as relative volumes.^74^  *P*-values were derived from posterior simulations using the relationship that an X% confidence interval that did not include the null value (relative volume = 1) was equivalent to *P* < 1 − X/100.

## Results

### Participant characteristics

**Figure 1:**
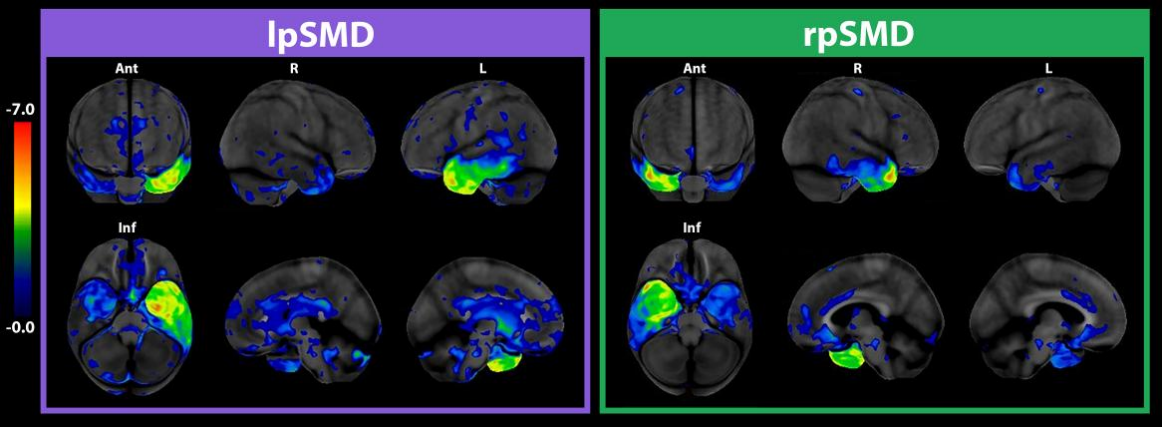
**Pattern of FDG-PET hypometabolism in SMD variants.** The CortexIDs show bilateral yet markedly asymmetric hypometabolism of the anterolateral and medial temporal lobes. The hypometabolism is left lateralized in lpSMD and right lateralized in rpSMD. The pattern of hypometabolism in the predominantly involved hemisphere is reminiscent of a ‘boxing glove’. Ant, anterior; Inf, inferior; L, left; lpSMD, left-predominant semantic dementia; R, right; rpSMD, right-predominant semantic dementia.

Of the 35 SMD participants, 25 (71%) were categorized as lpSMD and 10 (29%) as rpSMD based on the finding of predominant ‘boxing glove’ pattern of left or right anterior and medial temporal lobe hypometabolism, respectively (Fig. 1). Demographic, clinical, and imaging characteristics are shown in Table 1. There were no significant differences (*P* ≥ 0.05) in sex, years of education, and age at MRI scan amongst the control (*n* = 15), lpSMD, and rpSMD groups. Clinically, both lpSMD and rpSMD had similar distribution of handedness, as well as median age at onset and disease duration at baseline evaluation. The pharmacological history of SMD participants is shown in Supplementary Table 1. The lpSMD group had lower scores on the Frontal Assessment Battery (median scores: 15/18 versus 18/18, *P* = 0.03), with the lower scores apparently driven by worse performance on the similarities (conceptualization) subtest of the test (median scores: 2/3 in lpSMD versus 3/3 in rpSMD, *P* = 0.07). On the other hand, the rpSDM group recognized fewer famous faces (median scores: 3/10 in rpSMD versus 9/10 in lpSMD, *P* = 0.002), which is suggestive of prosopagnosia. In terms of speech-language performances at baseline, the lpSMD group had lower median Western Aphasia Battery-Aphasia Quotient scores (86/100 versus 93/100, *P* = 0.04), suggestive of more language impairment. An initial assessment of hippocampal volumes showed smaller total hippocampal (given by the sum of left and right whole hippocampal volumes) (*P* = 0.009) and right whole hippocampal (*P* < 0.001) volumes in rpSMD than lpSMD.

**Table 1:** Demographic, clinical, and neuroimaging characteristics of participants

Characteristic	Control (*n* = 15)	lpSMD (*n* = 25)	rpSMD (*n* = 10)	*P*-value
Female	10 (67%)	13 (52%)	8 (80%)	0.28
Education, years	14 (13, 16)	16 (14, 16)	16 (13, 18)	0.41
Age at MRI, years	60 (56, 64)	64 (58, 69)	68 (63, 71)	0.05
Handedness				0.65
Right		21 (84%)	9 (90%)	
Left		2 (8%)	0 (0%)	
Ambidextrous		2 (8%)	1 (10%)	
Age at onset, years		61 (54, 66)	62 (58, 68)	0.53
Disease duration, years		3 (2, 5)	4 (2, 5)	0.71
***Clinical/neuropsychological tests***				
FAB (/18)		15 (14, 17)	18 (16, 18)	**0**.**03**
FAB similarities (/3)		2 (0, 3)	3 (2, 3)	0.07
FAB Luria (/3)		3 (3, 3)	3 (3, 3)	>0.99
UPDRS III (/148)		0 (0,2)	2 (0,4)	0.20
WAB praxis (/60)		59 (57, 60)	60 (59, 60)	0.07
NPI (/36)		4 (2, 7)	7 (3, 13)	0.28
CBI-R (/180)		35 (18, 51)	40 (34, 61)	0.58
CBI-R abnormal behaviour (/24)		1 (0, 5)	1 (1, 7)	0.66
20-BAS (/20)		3 (1, 4)	2 (1, 8)	0.57
MoCA (/30)	26 (24, 28)	20 (18, 23)	24 (20, 26)	0.12^a^
Famous faces (/10)		9 (7, 10)	3 (1, 6)	**0**.**002**
Auditory battery (/30)		25 (21, 26)	21 (17, 25)	0.15
Pyramids & Palm trees (/52)		41 (33, 47)	41 (38, 42)	0.76
***Speech-language tests***				
WAB-AQ (/100)		86 (80, 93)	93 (88, 96)	**0**.**04**
SYDBAT naming (/30)		6 (3, 17)	11 (8, 12)	0.29
SYDBAT semantic association (/30)		19 (14, 22)	15 (14, 18)	0.17
Letter fluency (FAS)		25 (15, 33)	29 (18, 35)	0.37
BDAE repetition (/10)		8 (7, 10)	9 (8, 10)	0.25
***Neuroimaging features***				
Total intracranial volume, L	1.5 (1.4, 1.6)	1.5 (1.4, 1.7)	1.4 (1.4, 1.5)	0.22
Total hippocampal volume, mL	6.7 (6.4, 7.3)	5.7 (5.2, 6.4)	5.1 (4.8, 5.5)	**0**.**009**^a^
Left hippocampal volume, mL	3.3 (3.1, 3.6)	2.4 (2.2, 3.0)	2.8 (2.7, 2.9)	0.06^a^
Right hippocampal volume, mL	3.5 (3.2, 3.7)	3.3 (3.0, 3.5)	2.3 (2.2, 2.6)	**<0**.**001**^a^

Data are shown as count (%) or median (Q1, Q3). *P*-values are from Fisher’s exact test, Kruskal–Wallis test or Wilcoxon rank-sum test, as appropriate. *P*-values less than 0.05 are considered significant and are shown in bold.

20-BAS, 20-item Behavioural Assessment Scale; AQ, aphasia quotient; BDAE, Boston Diagnostic Aphasia Examination; CBI-R, Cambridge Behavioural Inventory-Revised; FAB, Frontal Assessment Battery; MoCA, Montreal Cognitive Assessment; NPI, Neuropsychiatric Inventory; SYDBAT, Sydney Language Battery; UPDRS, Unified Parkinson’s Disease Rating Scale; WAB, Western Aphasia Battery.

^a^Comparing semantic dementia groups.

### Volumetric analysis

**Figure 2:**
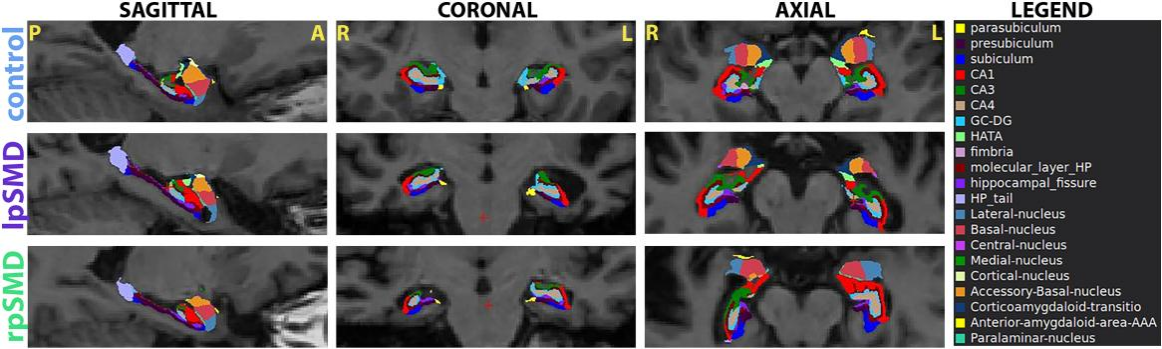
**Hippocampal subfield segmentation.** The figure shows representative T_1_-weighted MRI sagittal, coronal, and axial images for patients from the control, lpSMD, and rpSMD groups with overlying FreeSurfer 7-based segmentation of the subfields. Images are obtained using FreeSurfer Freeview tool. A, anterior; AAA, anterior amygdaloid area; GC-DG, dentate gyrus; HATA, hippocampal amygdaloid transition area; HP, hippocampus/hippocampal; L, left; lpSMD, left-predominant semantic dementia; P, posterior; R, right; rpSMD, right-predominant semantic dementia.

**Figure 3:**
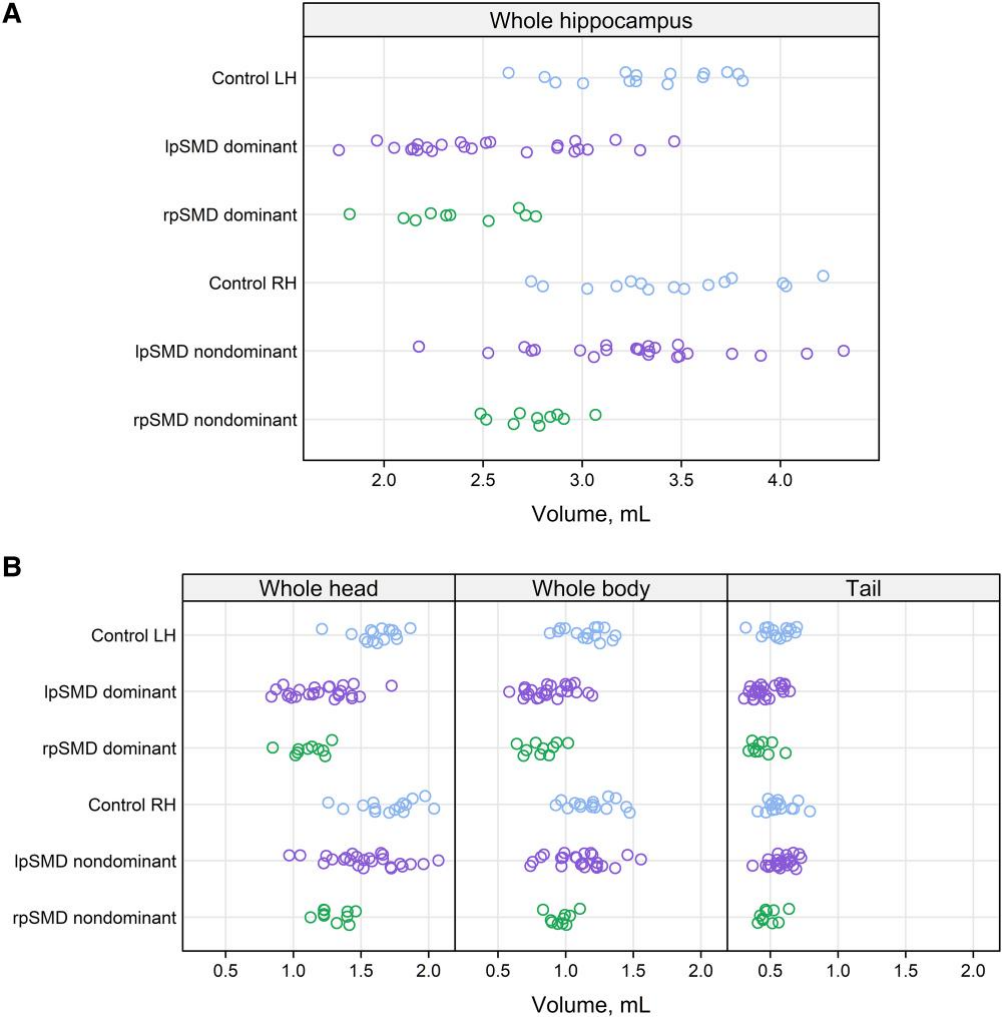
**Distribution of whole hippocampal and subregional volumes.** The scatter plots show the distribution of whole hippocampal volumes (**A**) and subregional (whole head, whole body, and tail) volumes (**B**) analysed. Volumes from the left and right hemispheres are shown for controls, whilst volumes from the dominant and non-dominant hemispheres are shown for the SMD variants (lpSMD and rpSMD). Dots represent a participant’s estimated hemispheric volume for the given hippocampal region. GC-ML-DG, dentate gyrus; HATA, hippocampal amygdaloid transition area; LH, left hemisphere; lpSMD, left-predominant semantic dementia; RH, right hemisphere; rpSMD, right-predominant semantic dementia.

**Figure 4:**
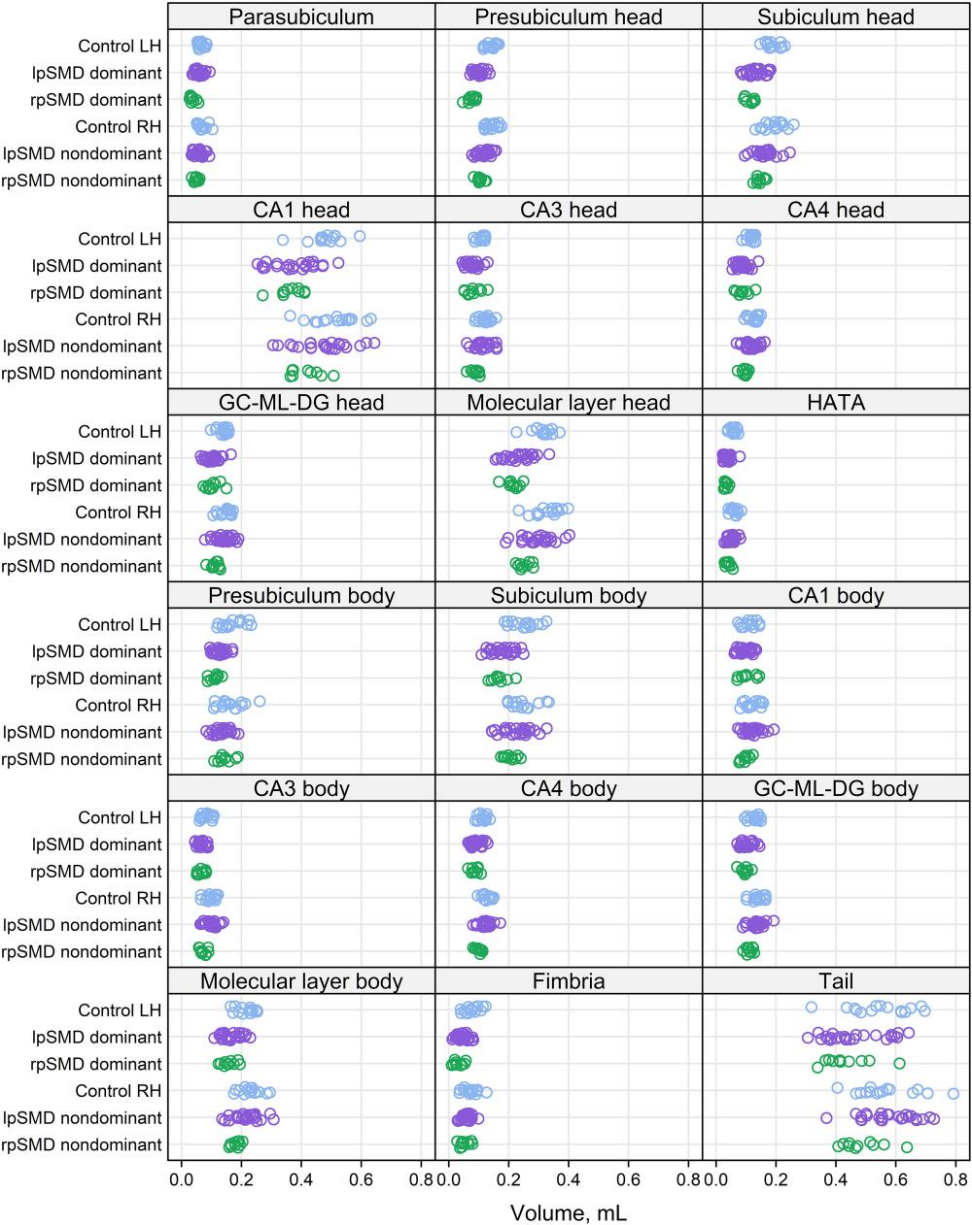
**Distribution of subfield volumes.** The scatter plots show the distribution of subfield volumes analysed. The subfields are separated based on the head or body localization. Volumes from the left and right hemispheres are shown for controls, whilst volumes from the dominant and non-dominant hemispheres are shown for the SMD variants (lpSMD and rpSMD). Dots represent a participant’s estimated hemispheric volume for the given hippocampal region. GC-ML-DG, dentate gyrus; HATA, hippocampal amygdaloid transition area; LH, left hemisphere; lpSMD, left-predominant semantic dementia; RH, right hemisphere; rpSMD, right-predominant semantic dementia.

FreeSurfer 7 segmentation of the subregions and subfields is shown in representative patients from the control, lpSMD, and rpSMD groups (Fig. 2). The distribution of whole hippocampal, subregion, and subfield volumes analysed are also shown in Figs 3 and 4.

#### Single-region whole hippocampal model

##### Dominant hemisphere

**Figure 5:**
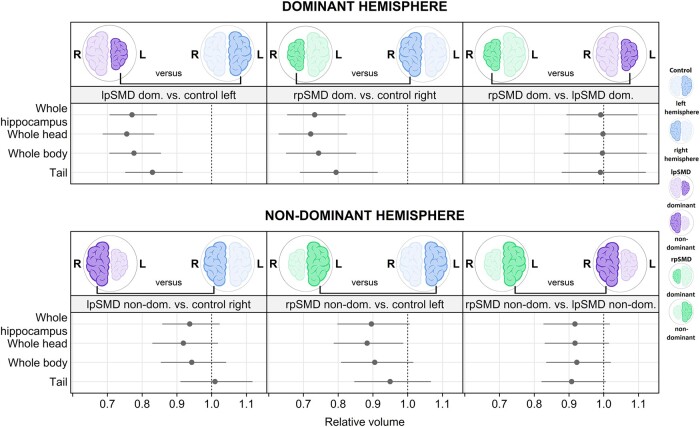
**Graphical summary of whole hippocampal and subregion models.** The forest plots show estimated decreases/increases in relative volume of the whole hippocampus and the subregions (whole head, whole body, and tail). Comparisons using the dominant hemispheres are shown in the top row, those using the non-dominant hemispheres are in the bottom row. The dots represent point estimates, and the lines represent 95% confidence intervals. Confidence intervals not crossing the line of null effect (vertical dotted line) are considered significant. Estimates are from Bayesian linear mixed-effects models and represent the posterior median, posterior 2.5th percentile and posterior 97.5th percentile. For easier interpretation, brain cartoons are shown in blue to represent controls, purple to represent lpSMD, and green to represent rpSMD. The smaller hemispheres represent the dominant side, whilst the bigger hemisphere represents the non-dominant side. dom, dominant; L, left; lpSMD, left-predominant semantic dementia; non-dom, non-dominant; R, right; rpSMD, right-predominant semantic dementia.

Considering the hippocampus as a whole (Fig. 5 and Supplementary Table 2), the dominant hemispheres of both lpSMD and rpSMD showed smaller whole hippocampal volumes (−23% for lpSMD and −27% for rpSMD) than the corresponding left and right hemispheres of controls (*P* < 0.001 for both). However, comparison of whole hippocampal volumes from the dominant hemispheres of both SMD variants did not show significant differences.

##### Non-dominant hemisphere

Regarding the non-dominant hemispheres, only a trend for smaller relative volumes in rpSMD compared with both controls (10% smaller volumes, *P* = 0.06) and lpSMD (8% smaller volumes, *P* = 0.09) was observed.

#### Three-region subregion model

##### Dominant hemisphere

At the head–body–tail subregion level (Fig. 5 and Supplementary Table 3), lpSMD showed 17–24% smaller head, body, and tail volumes of the dominant hemisphere compared with controls (*P* < 0.001 for all). Likewise, rpSMD showed 21–28% smaller volumes of all dominant hemisphere subregions relative to controls (*P* < 0.001 for head and body and *P* = 0.002 for tail subregion). Head, body, and tail volumes of both dominant hemispheres were comparable.

##### Non-dominant hemisphere

Concerning the non-dominant hemisphere, the subregion volumes of lpSMD did not differ from the controls. Conversely, the head subregion of rpSMD showed significant reductions in relative volumes versus control left hemisphere (−12%, *P* = 0.03), with a similar trend seen for the body subregion (−9%, *P* = 0.08). Comparison of subregion volumes from the non-dominant hemispheres of SMD variants showed trends for smaller rpSMD than lpSMD volumes at the level of head (−8%, *P* = 0.09) and tail subregions (−9%, *P* = 0.06).

##### Differences in effect sizes

We performed auxiliary analyses to determine whether there were significant differences amongst the effect sizes (Supplementary Table 4). Results showed that, compared with controls, the head subregion of the lpSMD dominant hemisphere demonstrated significantly greater volume loss than the tail subregion (*P* = 0.01), whilst only a trend was seen for the difference between the body and tail subregions (*P* = 0.05). A similar pattern was seen for rpSMD (head versus tail *P* = 0.02 and body versus tail *P* = 0.09). From the non-dominant hemisphere, both the head and body subregions of lpSMD showed significantly greater volume loss than the tail (*P* = 0.01 for head versus tail and *P* = 0.04 for body versus tail), although these did not differ from controls overall. There was also evidence for smaller head than tail volumes in the non-dominant rpSMD hemisphere (*P* = 0.06).

#### Eighteen-region subfield model

##### Dominant hemisphere

**Figure 6:**
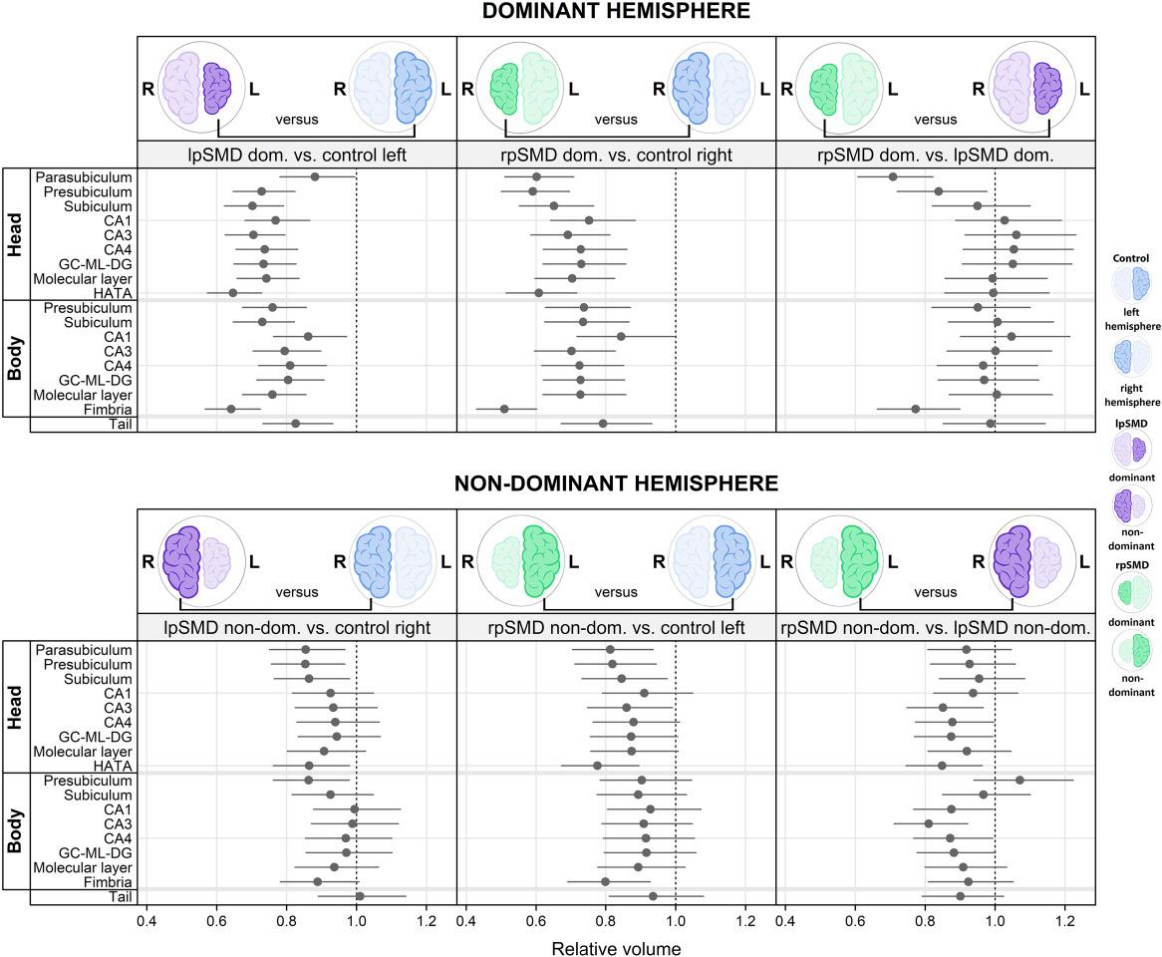
**Graphical summary of the subfield model.** The forest plots show estimated decreases/increases in relative volume of the subfields. Comparisons using the dominant hemispheres are shown in the top row, those using the non-dominant hemispheres are in the bottom row. The dots represent point estimates, and the lines represent 95% confidence intervals. Confidence intervals not crossing the line of null effect (vertical dotted line) are considered significant. Estimates are from Bayesian linear mixed-effects models and represent the posterior median, posterior 2.5th percentile and posterior 97.5th percentile. For easier interpretation, brain cartoons are shown in blue to represent controls, purple to represent lpSMD, and green to represent rpSMD. The smaller hemispheres represent the dominant side, whilst the bigger hemisphere represents the non-dominant side. CA, cornu ammonis; dom, dominant; GC-ML-DG, dentate gyrus; HATA, hippocampal–amygdaloid transition area; L, left; lpSMD, left-predominant semantic dementia; non-dom, non-dominant; R, right; rpSMD, right-predominant semantic dementia.

Results from the subfield analyses are shown in Fig. 6 and Supplementary Table 5. Compared with controls, the dominant hemisphere of lpSMD showed smaller volumes across *all* subfields (*P* = 0.02 for CA1 body, *P* = 0.04 for parasubiculum, *P* = 0.002 for hippocampal tail and *P* < 0.001 for all remaining subfields), with the greatest volume loss seen in the fimbria (−36%) and the HATA (−35%). The regions showing relatively more preserved volumes were the parasubiculum (−12%) and the CA1 from the body subregion (−14%). All other subfields showed about 17–30% smaller volumes relative to controls. Similarly, the dominant hemispheres of rpSMD showed smaller volumes of all subfields compared with controls (*P* < 0.001 for all, except for the CA1 body with *P* = 0.04 and hippocampal tail with *P* = 0.005). The greatest volume loss was again seen at the level of the fimbria (−49%), whereas the CA1 from the body subregion showed the smallest changes in volume (−16%). All the other subfields displayed decreases in volumes that ranged between −25% and −41%. Comparison of the dominant hemispheres showed smaller volumes of the parasubiculum (−29%) and fimbria (−23%) in rpSMD (*P* < 0.001 for both), as well as of the presubiculum from the head subregion (−16%, *P* = 0.02) versus lpSMD.

##### Non-dominant hemisphere

As for the non-dominant hemisphere, there were 14–15% smaller volumes of the parasubiculum (*P* = 0.01), presubiculum (from the head) and presubiculum (from the body) (*P* = 0.01 and *P* = 0.02, respectively), subiculum (from the head) (*P* = 0.02) and HATA (*P* = 0.02) in lpSMD relative to controls. There was also a trend for smaller volumes of the fimbria (−11%, *P* = 0.06). Interestingly, the non-dominant hemisphere of rpSMD showed 14–22% smaller volumes of a greater number of regions, including the parasubiculum (*P =* 0.004), presubiculum (*P* = 0.005), subiculum (*P* = 0.02), CA3 (*P* = 0.04) and HATA (*P* < 0.001) all from the head subregion, as well as the fimbria from the body subregion (*P* = 0.002). There were also trends for ∼12–13% smaller volumes of the other subfields from the head subregion, such as the CA4 (*P* = 0.07), dentate gyrus (*P* = 0.06), and molecular layer (*P* = 0.06). Comparison of non-dominant hemispheres showed volumes decreased by 15% in CA3 (*P* = 0.01), 12% in CA4 (*P* = 0.04), 13% in dentate gyrus (*P* = 0.04) and 15% in HATA (*P* = 0.01) (all from the head subregion), as well as 12% in CA1 (*P* = 0.04), 19% in CA3 (*P* = 0.002) and 13% in CA4 (*P* = 0.04) (all from the body subregion) in rpSMD versus lpSMD.

## Discussion

The involvement of the hippocampus in SMD is now generally accepted. In this study, we explored in detail the possible involvement of the hippocampal subregions and subfields in SMD variants and specifically assessed the differential patterns of volume loss between the dominant and non-dominant hemispheres in lpSMD and rpSMD and how these differed from controls. Specifically, we found that (i) at the whole hippocampal level, only the dominant hemisphere showed relative volume loss in lpSMD, whilst there was evidence for greater bilateral volume loss in rpSMD, with whole hippocampal volume loss in both dominant and non-dominant hemispheres; (ii) at the subregion level, all subregions (i.e. head, body, and tail) from the dominant hemispheres were affected in both lpSMD and rpSMD; however, there was also evidence of bilateral involvement of all subregions in rpSMD and a possible anteroposterior progression of volume loss (from head to body to tail); and (3) at the subfield level, the dominant hemispheres of both lpSMD and rpSMD showed volume loss across all subfields independent of the subregional localization (subfields in the head versus subfields in the body), but a greater number of subfields, particularly from the head subregion, from both dominant and non-dominant hemispheres were more affected in rpSMD than lpSMD. The subfields that showed consistent involvement in both dominant and non-dominant hemispheres and in both SMD variants were the parasubiculum, presubiculum, subiculum, and HATA all from the head subregions and the fimbria from the body subregion. Taken together, these suggest that hippocampal volume loss in SMD may start in one hemisphere and then progress to involve the other hemisphere and that these changes in whole hippocampal volumes are driven by changes in all subregions and subfields. However, the pattern of progression appears to be more subregion dependent (anteroposterior gradient) than subfield dependent. Whilst it needs to be validated in a longitudinal study, this suggests that subregion volumes might be a better biomarker for disease diagnosis and progression than whole hippocampal or individual subfield volumes.

In this study, we provide more evidence of involvement of the hippocampus in SMD, which corroborates previous findings.^23,53,57,75^ The cardinal symptom of SMD is impaired semantic memory that translates into loss of conceptual knowledge.^6,7^ Semantic memory is associated with noetic (knowing) consciousness that allows for introspective awareness of both internal and external worlds.^2^ The anterior temporal lobe, also called the temporopolar region, is a pivotal structure positioned at the confluence of auditory, visual, and limbic pathways whose degeneration is widely associated with verbal and non-verbal conceptual knowledge loss.^7,24^ A left-lateralization pattern of involvement is usually seen in lpSMD,^29^ whereas there is bilateral involvement of the anterior temporal pole with rightward predominance in rpSMD or right temporal SMD.^9,76^ However, several authors have argued that the loss of conceptual knowledge only arises following bilateral damage to the anterior temporal lobes.^77,78^ It has also been suggested that SMD starts in one hemisphere and naturally progresses to involve the contralateral hemisphere and that the two variants eventually merge as one.^27^ The handful of studies investigating hippocampal volumes in SMD reported conflicting results. Some showed bilateral involvement of the hippocampus in SMD, with left- or right-lateralization that typically followed the pattern seen in the anterolateral temporal lobe.^23,59,60^ Some authors reported early involvement of the right hippocampus in svPPA or lpSMD,^58^ yet others investigating similar hippocampal volumes in lpSMD detected no involvement of the right hippocampus.^53^ This is only partially in agreement with our findings. From our analyses, we found that when considering the overall hippocampus, there is a mostly unilateral involvement in lpSMD, whilst there is a more bilateral right-predominant involvement in rpSMD that appears to be driven mostly by changes in the head subregion. This is consistent with the clinical presentation, since the left hippocampus is said to be related to verbal memory, whilst the right hippocampus is associated with spatial and navigational memory.^43-45^ The unilateral hippocampal involvement in lpSMD and the bilateral involvement in rpSMD can be explained by different factors. One explanation could be that lpSMD and rpSMD are two different clinicopathological entities with dissimilar disease mechanisms. From a neuropathological standpoint, the anterior temporal lobe is highly susceptible to transactive response DNA-binding protein 43 (TDP-43) proteinopathy, particularly one that is characterized by the presence of long dystrophic neurites within the superficial cortical laminae and is referred to as frontotemporal lobar degeneration with TDP-43 type C pathology.^9,24,79-81^ Indeed, it has been reported that the presence of clinical symptoms of svPPA correctly predicts an underlying frontotemporal lobar degeneration with TDP-43 type C pathology with 80% sensitivity and 98% specificity.^81^ As with rpSMD, the same type of TDP-43 pathology is most frequently reported but it appears to account for less overall percentage of cases, with some cases caused by tauopathies like Pick’s disease, progressive supranuclear palsy, and globular glial tauopathy.^9,76,82^ Nonetheless, it is worth highlighting that some cases of the behavioural variant of frontotemporal dementia, which is caused by a more heterogeneous group of pathologies, are often mistaken for rpSMD.^82^ Another explanation for the bilateral involvement of the hippocampus in our study could be due to a more aggressive disease mechanism in the rpSMD, which quickly spreads to the contralateral hemisphere. However, it is also very likely that our lpSMD and rpSMD were evaluated at different stages of the disease. Although there were no differences in the reported disease duration at the time that our patients were seen at baseline, determining the exact time of disease onset could be more challenging in rpSMD than in lpSMD, as initial mild changes in behaviour or impairment of non-verbal domains can be subtle and less evident than the language impairment seen in lpSDM. It is possible that by the time our patients were diagnosed with rpSMD, the disease had already progressed to later stages compared with lpSMD. To support this theory is the finding that some subfields of the dominant hemisphere were more affected in rpSMD. Finally, another explanation could also be an increased vulnerability of the left hippocampus to normal aging, genetic factors, environmental factors, and different types of pathologies, including Alzheimer’s disease, since several studies have reported smaller volumes of the left hippocampus in cognitively normal young and older adults^83-86^ and in patients with varying degrees of Alzheimer’s disease pathology.^87,88^ Future studies investigating longitudinal changes across SMD could help shed light on the aetiology of this finding. However, some of our findings at the subregion and subfield levels might favour a true SMD effect.

Different from other studies, we assessed changes in subregion and subfield volumes in both SMD variants. We found that all subregions had smaller volumes in the dominant hemisphere, but only the head subregion was significantly affected in the non-dominant hemispheres. Damage to the anterior (head) hippocampus and its connections with cortical and subcortical structures has been closely linked to semantic memory impairment.^23,53^ It is possible that since encoding retrieval follows an anteroposterior gradient,^39^ impaired encoding by the anterior hippocampus could affect the acquisition and consolidation of semantic memories. The bilateral involvement of the head in both SMD variants is also consistent with the notion that unilateral damage to the hippocampus might not be sufficient to produce impaired semantic memory.^37^ We also found evidence for an anteroposterior gradient of atrophy as reported by other studies,^23,34,53,57^ whereby the greatest volume loss was in the anterior head subregion, less in the body, and least in the tail. Previous studies report that unilateral atrophy of the anterior hippocampus occurs in early cases of svPPA^53^ and spreads posteriorly as the disease progresses.^89^ Indeed, impairment of episodic memory as the disease progresses in SMD has been reported, possibly related to disruption of connections between the posterior hippocampus and parietal regions.^36,89,90^ Failure to update semantic memory can also lead to episodic memory problems.^91^

For our volumetric analysis, we separated the subfields based on their localization in the head or body subregion. We found that all subfields were affected in the dominant hemisphere in both lpSMD and rpSMD. However, in the non-dominant hemispheres, only a few subfields showed smaller volumes, most of which were in the head subregion. These findings suggest that changes in subfield volume also follow an anteroposterior gradient (head versus body localization) rather than the actual anatomical and functional division of the subfields. This was most evident when analysing the non-dominant hemispheres, as many subfields from the head subregion (more in rpSMD than in lpSMD) showed volume loss, whilst the same subfields in the body subregion did not. Overall, this suggests that the changes in hippocampal volumes in SMD are not necessarily subfield dependent but are instead more subregion dependent. Nonetheless, there were a few subfields that showed consistent involvement in both dominant and non-dominant hemispheres; these included the parasubiculum, presubiculum, subiculum, and HATA (all from the head subregion) and the fimbria (from the body subregion). It is possible that these subfields could represent the earliest regions affected in SMD. The presubiculum and subiculum have been formerly implicated in SMD.^57,58,60^ The few existing studies on hippocampal subfields in SMD did not assess changes in the parasubiculum, HATA, or fimbria since they were deemed too small to be segmented correctly.^58^ The condensed distribution of our data and the estimated relative volumes with their relatively narrow confidence intervals support the validity of our measurements. Moreover, the HATA and the fimbria are the two regions that often showed striking decreases in volumes in all our analyses and the involvement of the parasubiculum together with the presubiculum and subiculum that are known to be affected in SMD suggests consistency in our overall findings. Nonetheless, two previous studies had found consistent involvement of the CA1 subfield.^57,59^ Whilst we did find decreased volumes of the CA1, it was only in the dominant hemispheres where all subfields showed smaller volumes compared with controls and in the non-dominant hemisphere of rpSMD compared with lpSMD. There are several differences between the above-mentioned studies with predominant CA1 findings and ours: they only used patients with predominant language dysfunction clinically; they utilized a different segmentation method; and they measured the subfields as a whole, without splitting into head and body parts.

The parasubiculum, presubiculum and subiculum were formerly believed to form a structure called the ‘subicular cortex’, but recent findings now show that the three regions possess different intrinsic and extrinsic properties.^92^ The subiculum is the major output structure of the hippocampus; it receives input from the CA1 and in turn projects to the entorhinal cortex whilst also receiving reciprocal projections from the parasubiculum and presubiculum.^92^ The anterior subiculum projects to the medial band of the entorhinal cortex, which is then connected to the perirhinal cortex, insular and piriform cortex, amygdala, and basal ganglia.^39^ These networks are responsible for processing gist-like information, global spatial relations, and olfactory/gustatory output, amongst many other miscellaneous cortical inputs.^39^ On the other hand, the presubiculum and parasubiculum are involved in the visuospatial processing of scenic representations.^93,94^ The fimbria is a major route for both afferent and efferent fibres of the hippocampus, and it is associated with spatial learning and olfactory identification.^95,96^ Of note, the parasubiculum, presubiculum and fimbria that are all associated with spatial memory and visual processing of scenes all showed smaller volumes in rpSMD compared with lpSMD, which would be consistent with the more common symptoms of topographagnosia associated with this variant.^9^ Furthermore, the HATA is a structure that bridges the hippocampus to the amygdala and participates in the process of encoding and consolidation of memories of emotional events, fear conditioning, and extinction.^97^ This could explain the neurobehavioural symptoms typically reported, particularly in rpSMD participants. Involvement of all the above-mentioned subfields in the non-dominant hemisphere of lpSMD would also be in line with reports suggesting later development of behavioural changes and impairment in non-verbal domains of lpSMD.^27,58^ Furthermore, the dentate gyrus and the CA4 are other subfields reportedly affected in rpSMD and lpSMD.^58,60^ CA4 is additionally one of the first subfields reported to be affected in the contralateral hemisphere in svPPA in the middle stage of the disease.^58^ We found that the dentate gyrus and CA4, together with CA3 (all from the head subregion of the non-dominant hemisphere in rpSMD), showed smaller volumes compared with both lpSMD and controls. The non-dominant hemisphere of rpSMD also showed smaller volumes of the CA1 and CA3 in the body subregion compared with lpSMD. If we follow the logic of an anteroposterior gradient, these overall findings suggest that within the head subregion, the subiculum, parasubiculum, presubiculum and HATA are probably the first subfields affected, followed by CA3, CA4 and dentate, whilst the fimbria, CA1 and CA3 are probably the earliest subfields affected in the body subregion.

The strength of this study is the separate analysis of hippocampal volumes in both SMD variants. Another strength is the comparison between dominant and non-dominant hemispheres and the separation of subfields based on subregion localization, since these provided more comprehensive information on the different patterns of volume loss. However, this study is limited by the small number of rpSMD participants compared with lpSMD and by its cross-sectional nature. Future studies investigating longitudinal data could provide more insight into the actual progression of atrophy in SMD. We also acknowledge that hippocampal subfields lack internal contrast in these roughly 1-mm clinical resolution T_1_-weighted MRI, and thus the subfield segmentations mostly rely upon external atlas priors. Moreover, the resulting voxel size obtained with the MRI parameters used in this study is 0.2 mm^3^ greater than the 1.00 mm^3^ considered ideal voxel size for segmentation purposes. This study is also limited by the lack of T_2_-weighted MRI images to support FreeSurfer segmentation. Future work could address this by including higher resolution scans of the hippocampal region^73^ as well as the use of both T_1_- and T_2_-weighted images for segmentation. Furthermore, our cohort was predominantly Caucasian, which may somewhat limit the generalizability of the findings.

## Conclusion

In summary, the hippocampus along with all its subregions and subfields is affected in SMD. We found differences in subregion and subfield involvement in the two variants, with more subregions and subfields affected in number and to a greater degree in rpSMD compared with lpSMD. This can be explained by possible differences in disease stage at the time of evaluation since the clinical presentation in rpSMD can lead to delay in diagnosis. The pattern of volume loss shows hemispheric asymmetry based on the variant but generally follows an anteroposterior gradient with greater and apparently earlier involvement of the head subregion compared with the more posterior body and tail subregions. The pattern of involvement hence appears to be more subregion dependent than subfield dependent. Overall, the similar patterns of subregion and subfield involvement favour the notion that lpSMD and rpSMD are indeed two variants of the same disease that differ mostly based on the laterality of the hemisphere that presents with the earliest degeneration. Finally, our findings lay the foundation for future research to test the hypothesis that subregion volumes are more suitable biomarkers for disease diagnosis and progression than whole hippocampal volumes; whether subregion volumes truly outperform subfield volumes remains to be verified.

## Supplementary Material

fcae097_Supplementary_Data

## Data Availability

Data that support the findings of this study are available from the corresponding author (K.A.J.) upon request.
