# Supplementary material for: Volumetric analysis of hippocampal subregions and subfields in left and right semantic dementia
Source: Brain Commun. 2024 Mar 25;6(2):fcae097. doi: 10.1093/braincomms/fcae097 (PMC10988847; doi:10.1093/braincomms/fcae097)
Supplement: fcae097_Supplementary_Data [file fcae097_supplementary_data.pdf]

Supplementary Materials

Supplementary Table 1. Pharmacological history of participants.

**Medication Class**	**Left predominant SMD (*n*=25)**	**Right predominant SMD (*n*=10)**
Participants on long-term medications	*n*=14	*n*=5
Anti-Alzheimer's drugs	Donepezil (*n*=1)	None
Antianxiety/Hypnotics (benzodiazepines)	None	None
Anticonvulsants	Gabapentin (*n*=2)	Valproate (*n*=1)
Antidiabetics	Metformin (*n*=1)<br>Sulfonylurea (*n*=1)	None
Antidepressants	Desvenlafaxine (*n*=1) Mirtazapine (*n*=1)<br>Nortryptyline (*n*=1)<br>Sertraline (*n*=1)<br>Trazodone (*n*=1)	Citalopram (*n*=2)<br>Sertraline (*n*=1)<br>Trazodone (*n*=1)
Antihistamines	None	None
Antispasmodics	None	None
Anti-Parkinson's drugs	None	None
Cardiovascular drugs	ARB (*n*=1)<br>Beta-blocker (*n*=2)<br>Diuretic (*n*=2)	None
Chemotherapeutic agents	None	None
Corticosteroids	None	None
Narcotics	None	None
Non-benzodiazepine sedatives/hypnotics	Melatonin (*n*=1)<br>Zaleplon (*n*=1)	None
Statins	Statin (*n*=4)	None
Thyroid drugs	Levothyroxine (*n*=1)	None
Vitamins	B12 (*n*=2)	B12 (*n*=1)

Overall number of patients receiving each type of medication is reported stratified by semantic dementia variant. Only medications taken within one year prior to the research visit are reported. None of the participants from the control group were on any long-term medications.

Abbreviation: ARB = angiotensin receptor blocker; SMD = semantic dementia

Supplementary Table 2. Single-region model analysis of the left and right whole hippocampal volumes.

	Relative volume (95% CI)	P-value
Dominant hemisphere		
lpSMD relative to control left hemisphere	0.77 (0.71, 0.84)	**<0.001**
rpSMD relative to control right hemisphere	0.73 (0.65, 0.82)	**<0.001**
rpSMD relative to lpSMD	0.99 (0.89, 1.10)	0.86
Non-dominant hemisphere		
lpSMD relative to control left hemisphere	0.94 (0.86, 1.02)	0.14
rpSMD relative to control right hemisphere	0.90 (0.80, 1.00)	0.06
rpSMD relative to lpSMD	0.92 (0.83, 1.02)	0.09

Table of estimates, 95% confidence intervals, and *P*-values from the Bayesian hierarchical model. *P*-values are derived from posterior simulations using the relationship that an *X*% confidence interval that does not include the null value (relative volume of 1) is equivalent to $P < 1-X/100$. *P*-values <0.05 are considered significant and are shown in bold.

Abbreviations: lpSMD = left-predominant semantic dementia; rpSMD = right-predominant semantic dementia

Supplementary Table 3. Three-region model analysis of the left and right subregions.

Region	Hemisphere	Contrast	Relative volume (95% CI)	P-value
Whole head	Dominant	lpSMD relative to control left	0.76 (0.69, 0.83)	**<0.001**
		rpSMD relative to control right	0.72 (0.63, 0.82)	**<0.001**
		rpSMD relative to lpSMD dominant	1.00 (0.89, 1.12)	0.97
	Nondominant	lpSMD relative to control right	0.92 (0.83, 1.02)	0.10
		rpSMD relative to control left	0.88 (0.79, 0.99)	**0.03**
		rpSMD relative to lpSMD nondominant	0.92 (0.83, 1.01)	0.09
Whole body	Dominant	lpSMD relative to control left	0.78 (0.71, 0.85)	**<0.001**
		rpSMD relative to control right	0.74 (0.65, 0.85)	**<0.001**
		rpSMD relative to lpSMD dominant	1.00 (0.89, 1.12)	0.95
	Nondominant	lpSMD relative to control right	0.94 (0.85, 1.04)	0.24
		rpSMD relative to control left	0.91 (0.81, 1.02)	0.08
		rpSMD relative to lpSMD nondominant	0.92 (0.84, 1.02)	0.11
Tail	Dominant	lpSMD relative to control left	0.83 (0.75, 0.92)	**<0.001**
		rpSMD relative to control right	0.79 (0.69, 0.91)	**0.002**
		rpSMD relative to lpSMD dominant	0.99 (0.88, 1.12)	0.87
	Nondominant	lpSMD relative to control right	1.01 (0.91, 1.12)	0.85
		rpSMD relative to control left	0.95 (0.85, 1.07)	0.37
		rpSMD relative to lpSMD nondominant	0.91 (0.82, 1.00)	0.06

Table of estimates, 95% confidence intervals, and *P*-values from the Bayesian hierarchical model. *P*-values are derived from posterior simulations using the relationship that an *X*% confidence interval that does not include the null value (relative volume of 1) is equivalent to $P < 1-X/100$. *P*-values <0.05 are considered significant and are shown in bold.

Abbreviations: lpSMD = left-predominant semantic dementia; rpSMD = right-predominant semantic dementia

Supplementary Table 4. Comparison of "effect sizes" across head, body, and tail.

	Head minus body (95% CI)	P	Head minus tail (95% CI)	P	Body minus tail (95% CI)	P
Dominant hemisphere						
lpSMD relative to control left hemisphere	-0.02 (-0.07, 0.02)	0.39	-0.07 (-0.13, -0.01)	**0.01**	-0.05 (-0.11, -0.00)	0.05
rpSMD relative to control right hemisphere	-0.02 (-0.08, 0.03)	0.40	-0.07 (-0.15, -0.01)	**0.02**	-0.05 (-0.12, 0.01)	0.09
rpSMD relative to lpSMD	0.00 (-0.06, 0.07)	0.98	0.01 (-0.06, 0.07)	0.81	0.01 (-0.07, 0.07)	0.83
Nondominant hemisphere						
lpSMD relative to control left hemisphere	-0.02 (-0.08, 0.03)	0.40	-0.09 (-0.16, -0.02)	**0.01**	-0.07 (-0.14, -0.00)	**0.04**
rpSMD relative to control right hemisphere	-0.02 (-0.09, 0.03)	0.44	-0.06 (-0.15, 0.00)	0.06	-0.04 (-0.12, 0.02)	0.18
rpSMD relative to lpSMD	-0.00 (-0.07, 0.05)	0.88	0.01 (-0.05, 0.07)	0.71	0.01 (-0.04, 0.08)	0.62

P-values <0.05 are considered significant and are shown in bold.

Abbreviations: lpSMD = left-predominant semantic dementia; rpSMD = right-predominant semantic dementia

Supplementary Table 5. Eighteen-region model analysis of subfield volumes.

	Comparison	Region	Relative volume (95% CI)	P-value
1	lpSMD dominant relative to control left	CA1_body	0.86 (0.76, 0.97)	**0.02**
2	lpSMD dominant relative to control left	CA1_head	0.77 (0.68, 0.87)	**<0.001**
3	lpSMD dominant relative to control left	CA3_body	0.79 (0.70, 0.90)	**<0.001**
4	lpSMD dominant relative to control left	CA3_head	0.71 (0.62, 0.80)	**<0.001**
5	lpSMD dominant relative to control left	CA4_body	0.81 (0.72, 0.91)	**<0.001**
6	lpSMD dominant relative to control left	CA4_head	0.74 (0.65, 0.83)	**<0.001**
7	lpSMD dominant relative to control left	fimbria	0.64 (0.57, 0.73)	**<0.001**
8	lpSMD dominant relative to control left	GC_ML_DG_body	0.80 (0.71, 0.91)	**<0.001**
9	lpSMD dominant relative to control left	GC_ML_DG_head	0.73 (0.65, 0.83)	**<0.001**
10	lpSMD dominant relative to control left	HATA	0.65 (0.57, 0.73)	**<0.001**
11	lpSMD dominant relative to control left	Hippocampal_tail	0.83 (0.73, 0.93)	**0.002**
12	lpSMD dominant relative to control left	molecular_layer_HP_body	0.76 (0.67, 0.86)	**<0.001**
13	lpSMD dominant relative to control left	molecular_layer_HP_head	0.74 (0.66, 0.84)	**<0.001**
14	lpSMD dominant relative to control left	parasubiculum	0.88 (0.78, 1.00)	**0.04**
15	lpSMD dominant relative to control left	presubiculum_body	0.76 (0.67, 0.86)	**<0.001**
16	lpSMD dominant relative to control left	presubiculum_head	0.73 (0.65, 0.82)	**<0.001**
17	lpSMD dominant relative to control left	subiculum_body	0.73 (0.65, 0.82)	**<0.001**
18	lpSMD dominant relative to control left	subiculum_head	0.70 (0.62, 0.79)	**<0.001**
19	rpSMD dominant relative to control right	CA1_body	0.84 (0.72, 1.00)	**0.04**
20	rpSMD dominant relative to control right	CA1_head	0.75 (0.64, 0.88)	**<0.001**
21	rpSMD dominant relative to control right	CA3_body	0.70 (0.59, 0.83)	**<0.001**
22	rpSMD dominant relative to control right	CA3_head	0.69 (0.58, 0.81)	**<0.001**
23	rpSMD dominant relative to control right	CA4_body	0.72 (0.62, 0.85)	**<0.001**
24	rpSMD dominant relative to control right	CA4_head	0.73 (0.62, 0.86)	**<0.001**
25	rpSMD dominant relative to control right	fimbria	0.51 (0.43, 0.60)	**<0.001**
26	rpSMD dominant relative to control right	GC_ML_DG_body	0.73 (0.62, 0.85)	**<0.001**
27	rpSMD dominant relative to control right	GC_ML_DG_head	0.73 (0.62, 0.86)	**<0.001**
28	rpSMD dominant relative to control right	HATA	0.61 (0.51, 0.72)	**<0.001**
29	rpSMD dominant relative to control right	Hippocampal_tail	0.79 (0.67, 0.93)	**0.005**
30	rpSMD dominant relative to control right	molecular_layer_HP_body	0.73 (0.62, 0.86)	**<0.001**
31	rpSMD dominant relative to control right	molecular_layer_HP_head	0.70 (0.60, 0.83)	**<0.001**
32	rpSMD dominant relative to control right	parasubiculum	0.60 (0.51, 0.71)	**<0.001**
33	rpSMD dominant relative to control right	presubiculum_body	0.74 (0.63, 0.87)	**<0.001**
34	rpSMD dominant relative to control right	presubiculum_head	0.59 (0.50, 0.70)	**<0.001**
35	rpSMD dominant relative to control right	subiculum_body	0.73 (0.62, 0.87)	**<0.001**
36	rpSMD dominant relative to control right	subiculum_head	0.65 (0.55, 0.77)	**<0.001**
37	rpSMD dominant relative to lpSMD dominant	CA1_body	1.05 (0.90, 1.21)	0.54
38	rpSMD dominant relative to lpSMD dominant	CA1_head	1.03 (0.89, 1.19)	0.71
39	rpSMD dominant relative to lpSMD dominant	CA3_body	1.00 (0.86, 1.16)	0.99
40	rpSMD dominant relative to lpSMD dominant	CA3_head	1.06 (0.91, 1.23)	0.44
41	rpSMD dominant relative to lpSMD dominant	CA4_body	0.97 (0.83, 1.12)	0.66
42	rpSMD dominant relative to lpSMD dominant	CA4_head	1.05 (0.91, 1.22)	0.49
43	rpSMD dominant relative to lpSMD dominant	fimbria	0.77 (0.66, 0.90)	**<0.001**
44	rpSMD dominant relative to lpSMD dominant	GC_ML_DG_body	0.97 (0.84, 1.12)	0.68
45	rpSMD dominant relative to lpSMD dominant	GC_ML_DG_head	1.05 (0.91, 1.22)	0.51
46	rpSMD dominant relative to lpSMD dominant	HATA	0.99 (0.86, 1.15)	0.94
47	rpSMD dominant relative to lpSMD dominant	Hippocampal_tail	0.99 (0.85, 1.14)	0.86
48	rpSMD dominant relative to lpSMD dominant	molecular_layer_HP_body	1.01 (0.87, 1.16)	0.95

	Comparison	Region	Relative volume (95% CI)	P-value
49	rpSMD dominant relative to lpSMD dominant	molecular_layer_HP_head	0.99 (0.86, 1.15)	0.93
50	rpSMD dominant relative to lpSMD dominant	parasubiculum	0.71 (0.61, 0.82)	**<0.001**
51	rpSMD dominant relative to lpSMD dominant	presubiculum_body	0.95 (0.82, 1.10)	0.51
52	rpSMD dominant relative to lpSMD dominant	presubiculum_head	0.84 (0.72, 0.98)	**0.02**
53	rpSMD dominant relative to lpSMD dominant	subiculum_body	1.01 (0.87, 1.17)	0.92
54	rpSMD dominant relative to lpSMD dominant	subiculum_head	0.95 (0.82, 1.10)	0.48
55	lpSMD nondominant relative to control right	CA1_body	0.99 (0.88, 1.13)	0.93
56	lpSMD nondominant relative to control right	CA1_head	0.93 (0.82, 1.05)	0.22
57	lpSMD nondominant relative to control right	CA3_body	0.99 (0.87, 1.12)	0.86
58	lpSMD nondominant relative to control right	CA3_head	0.93 (0.82, 1.06)	0.28
59	lpSMD nondominant relative to control right	CA4_body	0.97 (0.85, 1.10)	0.63
60	lpSMD nondominant relative to control right	CA4_head	0.94 (0.83, 1.06)	0.33
61	lpSMD nondominant relative to control right	fimbria	0.89 (0.78, 1.01)	0.06
62	lpSMD nondominant relative to control right	GC_ML_DG_body	0.97 (0.86, 1.10)	0.63
63	lpSMD nondominant relative to control right	GC_ML_DG_head	0.94 (0.83, 1.07)	0.36
64	lpSMD nondominant relative to control right	HATA	0.86 (0.76, 0.98)	**0.02**
65	lpSMD nondominant relative to control right	Hippocampal_tail	1.01 (0.89, 1.14)	0.88
66	lpSMD nondominant relative to control right	molecular_layer_HP_body	0.94 (0.82, 1.06)	0.30
67	lpSMD nondominant relative to control right	molecular_layer_HP_head	0.91 (0.80, 1.03)	0.12
68	lpSMD nondominant relative to control right	parasubiculum	0.85 (0.75, 0.97)	**0.01**
69	lpSMD nondominant relative to control right	presubiculum_body	0.86 (0.76, 0.98)	**0.02**
70	lpSMD nondominant relative to control right	presubiculum_head	0.85 (0.76, 0.97)	**0.01**
71	lpSMD nondominant relative to control right	subiculum_body	0.93 (0.82, 1.05)	0.22
72	lpSMD nondominant relative to control right	subiculum_head	0.86 (0.76, 0.98)	**0.02**
73	rpSMD nondominant relative to control left	CA1_body	0.93 (0.80, 1.07)	0.31
74	rpSMD nondominant relative to control left	CA1_head	0.91 (0.79, 1.05)	0.20
75	rpSMD nondominant relative to control left	CA3_body	0.91 (0.79, 1.05)	0.19
76	rpSMD nondominant relative to control left	CA3_head	0.86 (0.75, 0.99)	**0.04**
77	rpSMD nondominant relative to control left	CA4_body	0.92 (0.79, 1.05)	0.22
78	rpSMD nondominant relative to control left	CA4_head	0.88 (0.76, 1.01)	0.07
79	rpSMD nondominant relative to control left	fimbria	0.80 (0.69, 0.93)	**0.002**
80	rpSMD nondominant relative to control left	GC_ML_DG_body	0.92 (0.80, 1.06)	0.23
81	rpSMD nondominant relative to control left	GC_ML_DG_head	0.87 (0.76, 1.01)	0.06
82	rpSMD nondominant relative to control left	HATA	0.78 (0.67, 0.90)	**<0.001**
83	rpSMD nondominant relative to control left	Hippocampal_tail	0.94 (0.81, 1.08)	0.36
84	rpSMD nondominant relative to control left	molecular_layer_HP_body	0.89 (0.78, 1.03)	0.11
85	rpSMD nondominant relative to control left	molecular_layer_HP_head	0.87 (0.76, 1.01)	0.06
86	rpSMD nondominant relative to control left	parasubiculum	0.81 (0.70, 0.94)	**0.004**
87	rpSMD nondominant relative to control left	presubiculum_body	0.90 (0.78, 1.05)	0.16
88	rpSMD nondominant relative to control left	presubiculum_head	0.82 (0.71, 0.94)	**0.005**
89	rpSMD nondominant relative to control left	subiculum_body	0.89 (0.78, 1.03)	0.12
90	rpSMD nondominant relative to control left	subiculum_head	0.85 (0.73, 0.98)	**0.02**
91	rpSMD nondominant relative to lpSMD nondominant	CA1_body	0.88 (0.77, 0.99)	**0.04**
92	rpSMD nondominant relative to lpSMD nondominant	CA1_head	0.94 (0.82, 1.06)	0.32
93	rpSMD nondominant relative to lpSMD nondominant	CA3_body	0.81 (0.71, 0.92)	**0.002**
94	rpSMD nondominant relative to lpSMD nondominant	CA3_head	0.85 (0.75, 0.97)	**0.01**
95	rpSMD nondominant relative to lpSMD nondominant	CA4_body	0.87 (0.77, 0.99)	**0.04**
96	rpSMD nondominant relative to lpSMD nondominant	CA4_head	0.88 (0.77, 1.00)	**0.04**
97	rpSMD nondominant relative to lpSMD nondominant	fimbria	0.92 (0.81, 1.05)	0.23
98	rpSMD nondominant relative to lpSMD nondominant	GC_ML_DG_body	0.88 (0.78, 1.00)	0.05

	Comparison	Region	Relative volume (95% CI)	P-value
99	rpSMD nondominant relative to lpSMD nondominant	GC_ML_DG_head	0.87 (0.77, 0.99)	**0.04**
100	rpSMD nondominant relative to lpSMD nondominant	HATA	0.85 (0.74, 0.96)	**0.01**
101	rpSMD nondominant relative to lpSMD nondominant	Hippocampal_tail	0.90 (0.79, 1.02)	0.11
102	rpSMD nondominant relative to lpSMD nondominant	molecular_layer_HP_body	0.91 (0.80, 1.03)	0.14
103	rpSMD nondominant relative to lpSMD nondominant	molecular_layer_HP_head	0.92 (0.81, 1.05)	0.20
104	rpSMD nondominant relative to lpSMD nondominant	parasubiculum	0.92 (0.81, 1.05)	0.19
105	rpSMD nondominant relative to lpSMD nondominant	presubiculum_body	1.07 (0.94, 1.22)	0.31
106	rpSMD nondominant relative to lpSMD nondominant	presubiculum_head	0.93 (0.82, 1.06)	0.24
107	rpSMD nondominant relative to lpSMD nondominant	subiculum_body	0.97 (0.85, 1.10)	0.60
108	rpSMD nondominant relative to lpSMD nondominant	subiculum_head	0.95 (0.84, 1.09)	0.47

Table of estimates, 95% confidence intervals, and *P*-values from the Bayesian hierarchical model. *P*-values are derived from posterior simulations using the relationship that an *X*% confidence interval that does not include the null value (relative volume of 1) is equivalent to $P < 1-X/100$. *P*-values <0.05 are considered significant and are shown in bold.

Abbreviations: GC_ML_DG = dentate gyrus; lpSMD = left-predominant semantic dementia; rpSMD = right-predominant semantic dementia
